# Experiences of weight stigmatization in the Israeli healthcare system among overweight and obese individuals

**DOI:** 10.1186/s13584-022-00518-9

**Published:** 2022-01-31

**Authors:** Lena Sagi-Dain, Moran Echar, Naama Paska-Davis

**Affiliations:** 1 Department of Obstetrics and Gynecology, Carmel Medical Center, Genetics Institute, 7 Michal St, Haifa, Israel; 2 The Ruth and Bruce Rappaport Faculty of Medicine, Technion - Israel Institute of Technology, Haifa, Israel; 3 Ariel University, Ariel, Israel

**Keywords:** Avoidance of treatment, Health communication, Weight stigmatization

## Abstract

**Introduction:**

Weight stigmatization, a common phenomenon in the healthcare system, exerts numerous adverse consequences on patients' wellbeing. The objective of this study was to estimate the extent and characteristics of weight stigmatization in Israel, based on the reports of overweight and obese individuals.

**Methods:**

This study was performed by distribution of a cross-sectional open anonymous survey in social media platforms, targeting respondents with body mass index over 25 kg/m^2^. The questionnaire consisted of Likert-scale based as well as open-ended questions, evaluating the experience during past medical appointments. Our primary outcome was the prevalence of disrespectful treatment. Subgroup analysis was performed by various demographic characteristics.

**Results:**

Of the 1697 respondents, 59.0% reported frequent experiences of disrespectful approach, and 48.6% noted receiving suboptimal treatment related to excess weight. Insulting, insensitive and judgmental comments were noted by 58%, stemming from diverse healthcare disciplines, while 29.3% noted office equipment not suitable for overweight people. Avoidance of a needed medical appointment was reported by 40.5%, significantly associated with past adverse experiences of weight stigmatization in the healthcare system. The respondents offered numerous suggestions to improve the existing situation, including education of the medical personnel, thorough research of obesity, and establishment of specific guidelines for approach to patients with excess weight.

**Conclusion:**

Weight stigmatization is prevalent in Israeli healthcare system; thus, decreasing the rates of this phenomenon should be an important national goal. Formal education about the wide prevalence and adverse effects of weight stigmatization should be practiced by academic institutions, professional organizations, and regulatory bodies. Straightforward policies with continuing supervision should be endorsed by the healthcare system to prevent weight-based discrimination. Finally, appropriate-size equipment for obese patients should become one of the requirements for accreditation of medical centers and facilities.

**Supplementary Information:**

The online version contains supplementary material available at 10.1186/s13584-022-00518-9.

## Introduction

Weight stigmatization, defined as exhibition of prejudiced attitudes and discriminatory actions towards individuals based solely upon their weight and body size [1], is experienced by about 20–40% of obese individuals [2]. This phenomenon has been documented in numerous settings, including education, employment, and healthcare systems [3]. Negative attitudes of health practitioners towards patients with excess weight include stereotypes that these patients are lazy, lack self-control and willpower, unintelligent, are personally to blame for their weight, and noncompliant with treatment [4]. Whether explicit (i.e., conscious opinions and beliefs about a stigmatized group) or implicit (automatic and subconscious), these attitudes may noticeably interrupt the healthcare process [5]. Healthcare providers allocate less time for medical encounters with overweight and obese patients [6] and report lower respect for these patients [7], negatively affecting patient-centered communication and information giving. In addition, health practitioners tend to over-attribute medical symptoms and problems solely to the excess weight, missing the opportunity for early diagnosis and treatment for the underlying disorder [8]. For the patients, perception of weight stigma is associated with low self-esteem, depression and aggravation of eating disorders [6]. Furthermore, weight stigmatization in the healthcare system leads to decreased quality of communication, lower compliance and avoidance of medical appointments and screening tests [5]. Thus, it is crucial to investigate the prevalence of weight stigma in various healthcare systems, to define the extent, the nature and the factors associated with this phenomenon, and to introduce measures for its eradication and prevention.

According to Israeli national health interview survey, in 2019 the prevalence of overweight (defined as body mass index (BMI) of 25–29.9 kg/m^2^) in adults aged 20–64 years was estimated at 34.5%, ranging from 30.0% in women and up to 39.6% in men [9]. The rate of obesity (BMI over 30 kg/m^2^) is estimated as 24.1% (25.5% in women and 22.5% in men). Thus, excess weight involves about one half of all Israeli adults, exposing them to possible effects of weight stigma. Nevertheless, to our best knowledge, no previous evidence has been published examining the characteristics of weight stigma in Israeli healthcare system.

Thus, the objective of our study was to estimate the extent and characteristics of weight stigmatization in Israel from patients' points of view, and explore the factors associated with this phenomenon.

## Methods

This study was designed in conjunction with Checklist for Reporting Results of Internet E-Surveys (CHERRIES) guidelines [10]. As no validated tools were found on the specific topic, the questionnaire was based on clinical experience, correspondence with leading professionals and relevant updated literature. The introduction included an explanation that the target population is individuals with body mass index (BMI) over 25 kg/m^2^ and over the age of 18 years (i.e., the inclusion criteria). The informed consent process included explanation about the purpose and contents of the survey as well as the names of the investigators, prior to gaining access to the questionnaire. Potential participants were informed that the survey is anonymous, that its filling out takes about 5–10 min, and that the completion of the survey is not mandatory. In addition, the respondents were informed that the results of the questionnaire will be summarized and published in the form of a scientific article (Additional file 1: Document 1). Informed consent was enforced by instructing the respondents to access the questionnaire site only in case they agree to participate in the survey.

The questionnaire consisted of three main parts: first included demographic details (age, gender, marital status, ethnicity, education, weight, height, and BMI). The second part included Likert-scale based questions evaluating the experience during past medical appointments, including the quality of the received treatment and approach of the medical personnel. Several points included associated open-ended questions, mainly asking for examples. The third part of the questionnaire included questions evaluating the respondents' opinions concerning excess weight, and for their preferences regarding the approach of healthcare practitioners to the issue of overweight during medical appointments (not included in the current study). The questionnaire was administered in Hebrew; the translated document is available as a supplementary file to this report (Additional file 1: Document 1).

The questions were fixed, and no adaptive questioning processes were used. Non-response options were not included, since the only mandatory questions were weight and height (for BMI calculation and verification). Cookies were not used, and IP addresses of client computers were not collected, thus unique site visitor counts, as well as view rates and participation rates were not calculated. Since no personal identifying information was collected in the questionnaire, the survey was considered exempt from institutional review board approval (following a preliminary discussion with Carmel Medical Center Helsinki committee).

A convenience sample was achieved by distribution of a link to an internet-based cross-sectional open anonymous survey in social media platforms (mainly Facebook and Whatsapp). The link was initially distributed through authors' Facebook pages (LS—7000 followers, NP—7000 followers), and later shared by the readers on a voluntary basis, without any paid advertising. The responses to the survey were captured automatically using Google forms. Surveys were completed during September 2020. Following the survey completion, the data were downloaded and permanently deleted from Google forms.

Statistical Package for the Social Sciences (SPSS) v.24 software was used for most statistical analyses, while SAS version 9.4 was used for the PROC GENMOD procedure. Categorical variables were presented as numbers (percentage), and continuous variables with normal distribution (assessed by skewness test)—as means ± standard deviations. Answers to the second part of the questionnaire (description of past medical appointments—i.e., disrespectful approach, less optimal treatment, as well as insulting, insensitive and judgmental comments) were considered as positive when scored 3 (sometimes), 4 (in most cases), or 5 (all the time). Open-ended responses were inductively and independently coded by two investigators (LS, MI) into appropriate categories. Analysis of categorical variables were performed using Fisher's exact test or by relative risk (RR) with 95% confidence interval (CI) calculations. *P* value of < 0.05 was considered as statistically significant. Subgroup analysis was performed for age, gender, education, relationship status, and BMI categories: overweight (25–29.9 kg/m^2^), class I obesity (30–34.9 kg/m^2^), class II obesity (35–39.9 kg/m^2^), and class III obesity (BMI of 40 kg/m^2^ or greater) [11]. Finally, using logistic regression analysis, we have evaluated factors associated with one of the most alarming complications of weight stigmatization—avoidance of needed medical appointments. Our hypothesis was that experience of worse treatment and insulting attitude would increase such avoidance. Correlation between avoidance and the different covariates were assessed using generalized linear model with binominal distribution and log link function for each covariate. All variables yielding *p* value < 0.1 were entered into a multivariate model.

## Results

Overall, 1731 participants have filled out the questionnaire. Thirteen forms were excluded due to filling out less than 75% of the questions, and additional 21 questionnaires were not included due to BMI lower than 25. Baseline characteristics of the 1697 respondents are presented in Table 1.

**Table 1:** Baseline characteristics of the participants

The characteristic	Measure
Age (years)	36.6 ± 11.1 (median—35)
Gender	
Female	1629 (96.0%)
Male	68 (4.0%)
Relationship status	
Married/in a relationship	1156 (68.1%)
Single/divorced/widow/er	451 (31.9%)
Education status	
High	Bachelor’s degree—725 (42.7%) Master's degree—393 (23.2%) Doctorate—68 (4.0%)
Low	Certificate studies—238 (14.0%) High school education—236 (13.9%) Less than 12 years of schooling—15 (0.9%)
Body mass index (kg/m^2^)	36.5 + 6.6
Overweight (25–29.9 kg/m^2^)	267 (15.7%)
Class I obesity (30–34.9 kg/m^2^)	495 (29.2%)
Class II obesity (35–39.9 kg/m^2^)	479 (28.2%)
Class III obesity (BMI of 40 kg/m^2^ or greater)	456 (26.9%)

Recalling past medical treatment, the question “I felt that the approach of the medical staff is less respectful because of my excess weight” was annotated as “sometimes/in most cases/all the time” by 59.0% of the respondents. Frequent apprehension of “my overweight is causing the medical staff discomfort” was described in 43.8%, while the statement “because of my excess weight I receive less optimal treatment from the medical staff” was marked by 48.6% of the participants.

**Fig. 1:**
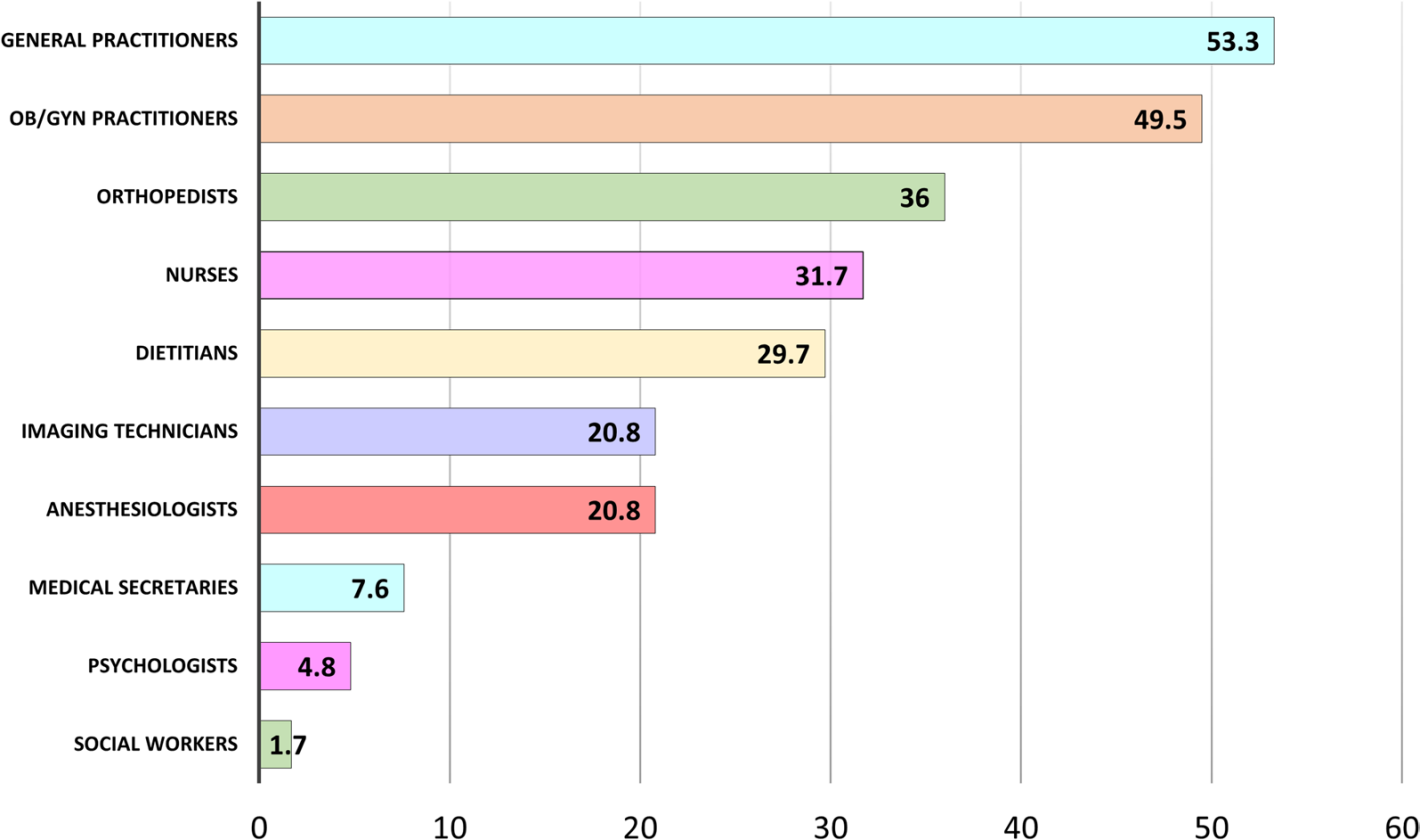
Medical staff members exerting insulting, insensitive or judgmental approach

Fifty eight percent of the responders have responded to the statement “During medical appointments I have experienced insulting, insensitive and judgmental approach related to my overweight” as “sometimes/in most cases/all the time”. Description of the offending medical staff members (distributed by highest to lowest rate) is presented in Fig. 1. Verbal descriptions of examples to insulting or disrespectful approach were inductively coded by the researchers into seven main categories, the most prominent of these was "referral to the overweight as the main issue, causing all other medical problems" (51.9%). Second most common category was humiliating and cynical comments, noted by 26.9% of the respondents. Complaints about technical difficulty to examine the patients were noted by 18.1% of the respondents, receiving no adequate treatment—in 14.2%, and explicit refusal to treat—in 8.4%. Descriptions of the harmful effects of obesity on beauty and relationships were noted in 10.0%, and statements perceived as frightening intimidations for the obesity effects on one's health—in 8.3%.

The statement “It happened that the office equipment was not suitable for overweight people” was annotated as “sometimes/in most cases/all the time” by 29.3% of the respondents. Examples of such equipment, given by 486 respondents, were small for size sphygmomanometers (61.1%), narrow chairs or chairs with handles (25.9%), narrow beds (7.8%), small shirts and robes (5.3%), small CT/MRI machines (3.7%), narrow weights (1.6%), short monitor straps (1.4%), narrow wheelchairs (1.0%), small toilet rooms (0.6%), short intravenous lines (0.4%), short speculums and gynecologic ultrasounds (0.4%), as well as narrow passages and doors (0.4%).

**Fig. 2:**
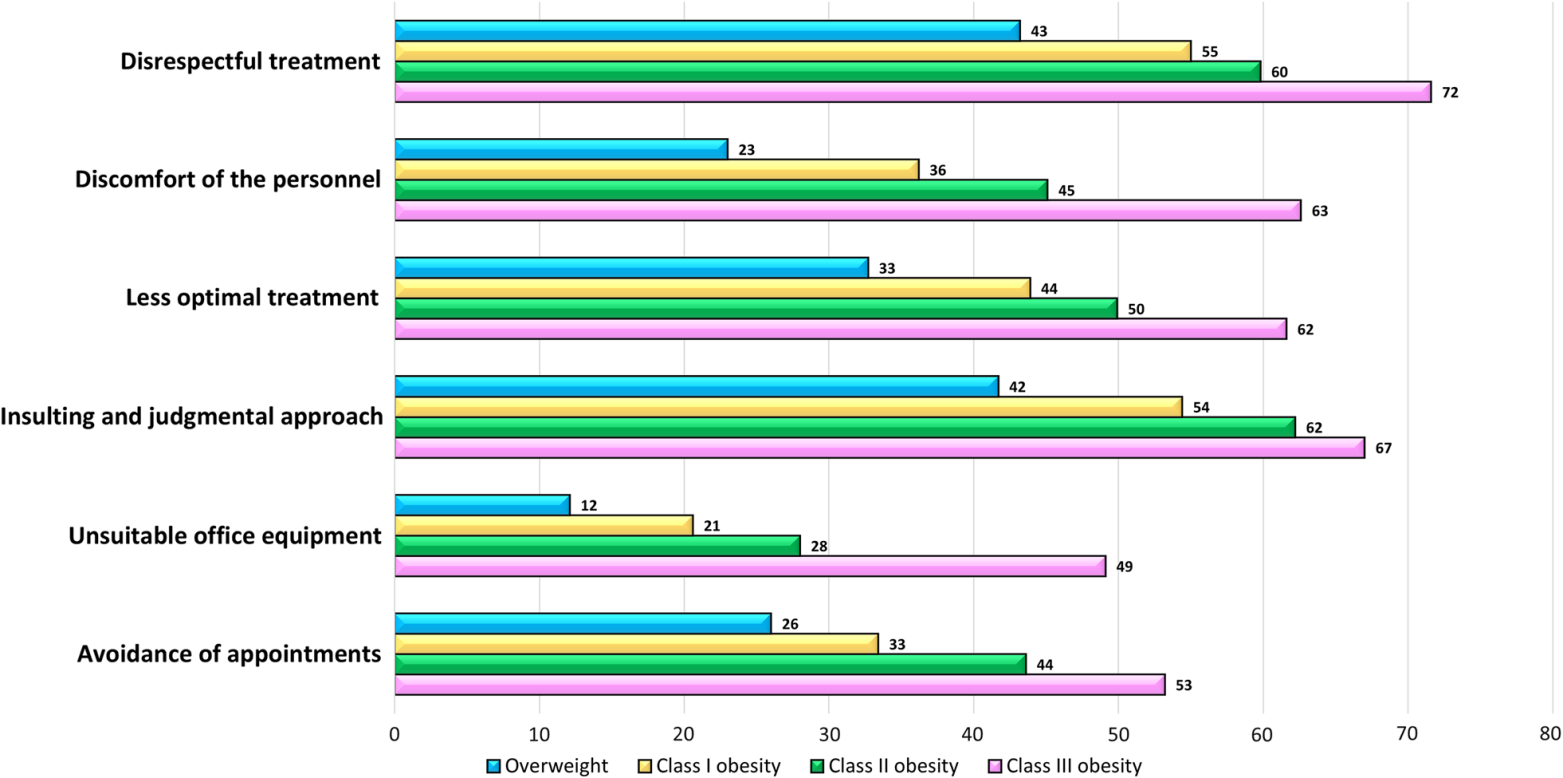
Rates of adverse feelings and experiences by weight categories. BMI categories: overweight (25–29.9 kg/m^2^), class I obesity (30–34.9 kg/m^2^), class II obesity (35–39.9 kg/m^2^), and class III obesity (BMI of 40 kg/m^2^ or greater)

Importantly, the question “Have you ever avoided a needed appointment with a doctor due to fear of disrespectful treatment because of excess weight?” was annotated as “sometimes/in most cases/all the time” by 40.5% of the respondents. Using a univariable analysis, all the examined adverse feelings and experiences, including disrespectful treatment, feeling of discomfort of the personnel, apprehension of less optimal treatment, insulting, insensitive and judgmental approach, unsuitable office equipment, as well as avoidance of needed medical appointments, were significantly correlated to increasing BMI (Fig. 2; Additional file 2: Table S2). In addition, in a univariate analysis, women experienced higher rates of disrespectful treatment (59.4% vs. 47.1, respectively, *p* = 0.045), higher appreciation of discomfort of the personnel due to overweight (44.2% vs. 31.3%, *p* = 0.044), perceptions of receiving less optimal treatment (49.2% vs. 33.8%, *p* = 0.012), and avoidance of needed appointments due to fear of disrespectful treatment (41.0% vs. 28.4%, *p* = 0.042), compared to male patients.

Correlation of various demographic and stigma parameters on avoidance of medical treatment is presented in Table 2. Using multivariate analysis for all parameters yielding a *p* value lower than 0.1, factors associated with avoidance of needed medical appointments were: class III obesity (adjusted RR (aRR) 1.22, 95%CI 1.01–1.47), apprehension of discomfort from the medical personnel (aRR 1.23, 95%CI 1.08–1.39), perception of less optimal treatment due to the excess weight (aRR 1.52, 95%CI 1.28–1.80), experience of insulting, insensitive and judgmental comments (aRR 1.91, 95%CI 1.48–2.46), disrespectful attitude (aRR 1.43, 95%CI 1.09–1.87) and unsuitable office equipment (aRR 1.11, 95%CI1.01–1.22).

**Table 2:** Correlation of various demographic and stigma parameters on avoidance of medical treatment

	Avoidance (n = 786)	No avoidance (n = 886)	RR 95%CI	*P* value	Adj RR 95%CI	*P* value
Age	35.6 ± 12.0	37.5 ± 10.2	0.98 (0.97–0.995)	0.0002	1.0 (0.99–1.002)	0.302
Gender						
Male	16 (26.7)	44 (73.3)	Ref			
Female	766 (47.7)	839 (52.3)	1.79 (1.17–2.7)	0.007	1.3 (0.88–1.93)	0.174
Family status						
Single	268 (50.2)	266 (49.8)	Ref			
In a relationship	518 (45.5)	620 (54.5)	0.91 (0.82–1.01)	0.07	0.97 (0.89–1.06)	0.495
Education						
Lower	243 (48.6)	257 (51.4)	Ref			
Higher	543 (46.3)	629 (53.7)	0.95 (0.85–1.06)	0.391		
BMI categories						
25–30	78 (30.2)	180 (69.8)	Ref			
30–35	179 (36.7)	309 (63.3)	1.21 (0.97–1.51)	0.084	0.96 (0.79–1.17)	0.721
35–40	240 (50.5)	235 (49.5)	1.67 (1.36–2.05)	< 0.0001	1.16 (0.96–1.40)	0.117
> 40	289 (64.1)	162 (35.9)	2.12 (1.74–2.58)	< 0.0001	1.22 (1.01–1.47)	0.035
Discomfort of the personnel						
No	228 (27.7)	594 (72.3)	Ref			
Yes	557 (66.2)	285 (33.8)	2.39 (2.11–2.69)	< 0.0001	1.23 (1.08–1.39)	0.002
Worse treatment						
No	160 (21.8)	573 (78.2)	Ref			
Yes	621 (66.8)	308 (33.2)	3.06 (2.65–3.54)	< 0.0001	1.52 (1.28–1.80)	< 0.0001
Insulting attitude						
No	87 (16.0)	458 (84,0)	Ref			
Yes	696 (62.3)	422 (37.7)	3.9 (3.2–4.8)	< 0.0001	1.91 (1.48–2.46)	< 0.0001
Inappropriate equipment						
No	403 (37.8)	662 (62.2)	Ref			
Yes	346 (64.6)	190 (35.4)	1.71(1.54–1.88)	< 0.0001	1.11 (1.01–1.22)	0.003
Disrespectful attitude						
No	84 (15.9)	444 (84.1)	Ref			
Yes	698 (61.4)	439 (38.6)	3.85 (3.15–4.72)	< 0.0001	1.43 (1.09–1.87)	0.010

Answers to the open-ended question "In your opinion, what can be done to improve the medical staff's attitude towards overweight and obese patients?", noted by 951 respondents, were inductively coded into 12 main issues, the most prominent of these is "raising the awareness to the existence and adverse effects of weight stigmatization" (Table 3).

**Table 3:** Prominent categories of answers to the question "In your opinion, what can be done to improve the medical staff's attitude towards overweight patients?”

The category	Number	%
Awareness of the existence and adverse effects of weight stigmatization	501	52.7
Instruction of medical personnel by courses, trainings, and workshops	184	19.3
Empathy, sensitivity, humanity, and respect	160	16.8
To see beyond the fat	103	10.8
To raise the issue of overweight during medical appointment only if relevant to the reason of the referral	68	7.2
Involvement of the healthcare system (supervisors, accessible ways to file complaints, penalties)	55	5.8
To ask the patients if it is okay to speak about their weight prior to raising the issue	50	5.3
Awareness to the fact that not every fat person is unhealthy	42	4.4
Awareness to the fact that fat people know they are fat, and have been trying to lose weight for years	40	4.2
To offer practical tools and not just tell to lose weight	34	3.6
Awareness to the fact that losing weight and sustaining lower weight is extremely hard	33	3.5
Not to refer to the issue of overweight	31	3.3
There is no hope	28	2.9

## Discussion

The results of the survey show that two thirds of overweight and obese patients are frequently faced with disrespectful approach related to excess weight, and encounter insulting, insensitive and judgmental comments. Furthermore, almost half of the respondents described avoiding a needed medical appointment due to fear of disrespectful treatment. Indeed, one of the main limitations of an online voluntary survey is selection bias, i.e., a higher urge to answer the questionnaire for respondents who have experienced disrespectful treatment in their past. Thus, the rates of adverse healthcare experiences noted in our study might be higher than in the general overweight and obese population. Nevertheless, our outcomes are in conjunction with previous studies examining the prevalence of weight stigma in healthcare system [12–14]. For instance, in a survey of 85 adults enrolled in behavioral weight loss program, 70.6% of participants reported at least one stigmatizing health care experience in the past year [12]. In another questionnaire-based study of 93 obese treatment-seeking adults, 89% reported experiencing inappropriate remarks from doctors [15]. Moreover, in a survey of 329 health professionals specializing in eating disorders, 56% reported observing their colleagues making negative comments about obese patients [16].

Weight stigmatization can manifest in different ways, including less patient-centered communication, less respectful approach, fewer positive affective communication and information providing, and allocating less time for medical appointments [5]. According to our survey, and in conjunction with previous evidence [3, 17], experiences of disrespectful approach spread across various medical professions. Main healthcare practitioners attributed to disrespectful care were general practitioners, gynecologists, specialists in orthopedics, and anesthesiologists (mainly during administration of epidural anesthesia). Thus, these specific health professionals must be aware of the adverse effect of weight stigma and adopt a particularly sensitive approach to patients with excess weight.

Most prominent category described in examples to insulting or disrespectful approach was referral of health practitioners to the excess weight as the main cause for any medical condition, even in clearly unrelated problems such as sinusitis, headache, etc. This approach was demonstrated in previous studies; for instance, in a survey of 161 adults attending dietetic outpatients clinics for obesity in Portsmouth, United Kingdom, 84% agreed that “weight is blamed for most medical problems' [18].

A fifth of the participants were upset due to health practitioners' complaints about technical difficulty to examine the patients. Even if the excess weight makes the test more cumbersome, medical personnel should be aware that unnecessary comments on the matter might contribute to patients' experience of weight stigma.

Office equipment not suitable for patients with excess weight was noted by 29.3% (and up to half of respondents with class III obesity). These numbers are in concordance with previous literature noting equipment failures for individuals with obesity [19, 20]. Thus, medical centers and clinics must supply proper size devices and facilities, including attention to wide-enough passages, doors, and rooms.

Avoidance of a needed medical appointment due to fear of disrespectful treatment because of the excess weight was reported by 40.5% of the respondents, reaching over 50% in patients with class III obesity. Not surprisingly, avoidance was correlated with adverse experiences of weight stigmatization in the healthcare system, with experience of disrespectful attitude and insulting, insensitive and judgmental comments yielding the highest relative risks. Our study is not the first evidence for the association between increased BMI to the tendency to delay or avoid health care [19, 21, 22]. For instance, obesity has been shown to impede breast, cervical and colorectal cancer screening tests [23, 24]. As individuals with excess weight are at higher risk for obesity-related diseases, healthcare avoidance for these patients might be associated with more severe health implications, highlighting the importance of a sensitive and respectful approach.

It was interesting to note that, in accordance with previous evidence [2], women noted higher rates of disrespectful treatment, compared to male patients. This might be related to increased levels of body dissatisfaction and internalized weight bias in female patients, as well as higher rates of weight-based stigma towards women in multiple domains, including employment, education, romantic relationships, and health care [25, 26].

The respondents offered numerous suggestions to improve the existing situation.

These intuitive suggestions parallel the recent recommendations of the joint international consensus statement to eliminate weight bias [4]. To our best knowledge, no guidelines are currently practiced in Israeli hospitals and healthcare organizations to combat weight stigma. Based on previous recommendations and the conclusions drawn from our survey, the following policy implications can be suggested to the healthcare system [4]. Formal education about the versatile etiology of obesity, as well as the wide prevalence and adverse effects of weight stigmatization, should be regularly practiced by academic institutions, professional organizations, and regulatory bodies. Standard curricula should be performed for medical students, residents and senior health practitioners, as well as other medical personnel including nurses, dietitians, etc. Straightforward policies for the prevention of weight-based discrimination should be endorsed by the healthcare system. Finally, appropriate-size equipment for obese patients should become one of the requirements for accreditation of medical centers and facilities.

Our study has several limitations. An inherent drawback of any survey is selection bias, caused by self-selection, or by distribution of the questionnaire in selected groups of individuals (i.e., Facebook pages significant containing focus on weight stigma). In addition, the questionnaire was distributed in Hebrew, compromising the generalizability of the results. Furthermore, several potential confounders were not addressed, such as health status of the participants and the frequency of medical appointments in the past year. Still, to our best knowledge, our study is the first survey aiming to characterize weight stigmatization in Israel, and the large number of responders is one of its main strengths.

## Conclusions

The issue of excess weight should be approached with empathy, sensitivity, humanity, and respect. Since weight stigma seems prevalent in Israel, decreasing the rates of this phenomenon in the healthcare system should be one of the important national goals. Medical personnel and healthcare system should be aware of the existence, the wide prevalence, and adverse consequences of weight stigmatization, and introduce mechanisms for education and supervision on this important topic.

## Supplementary Information

**Additional file 1.** The questionnaire.

**Additional file 2.** Univariate analysis of the correlation of weight categories to adverse feelings and experiences.

## Data Availability

The datasets used and/or analysed during the current study are available from the corresponding author on reasonable request.
