# Supplementary material for: Experiences of weight stigmatization in the Israeli healthcare system among overweight and obese individuals
Source: Isr J Health Policy Res. 2022 Jan 31;11:5. doi: 10.1186/s13584-022-00518-9 (PMC8802507; doi:10.1186/s13584-022-00518-9)
Supplement: Supplementary file 1 — Additional file 1. The questionnaire. [file 13584_2022_518_MOESM1_ESM.docx]

**Supplementary document 1 - The questionnaire.**

Dear participant,

This is Lena Sagi-Dain, a gynecologist and geneticist from Carmel Medical Center in Haifa.

I would appreciate your help in completing a short questionnaire, designed to assess the medical staff's attitude towards overweight patients, and the steps that can be taken for healthcare system improvement.

The questionnaire was designed for overweight people (BMI equals 25 or greater; the formula for calculating the BMI is the weight divided by the square of height in meters).

The questionnaire is **anonymous**, filling out the questionnaire takes around 5-10 minutes, and you can stop filling it out at any stage. The results of the questionnaire will be summarized and published in the form of a scientific article in the professional literature. The conclusions emerging from the analysis of the results will allow to adjust the existing policy and improve the approach of the medical staff to overweight people.

If you agree to fill out the questionnaire, please click on the image attached.

Thank you very much for your willingness to help!

**Please tell us some details about you**

**🖍 Age:** ________________

**🖍 Gender:**

❑       Male

❑       Female

❑       Other ____________

**🖍 Marital Status**

❑       Married

❑       In a relationship

❑       Single

❑       Divorced

❑       Widow/er

❑       Other

**🖍 Ethnicity:**

❑       Ashkenazi

❑       Sephardic

❑       Arab origin

❑       Ethiopia

❑       Bedouin

❑       Russian non-Jewish

❑       Other _______________

**🖍 Education**

❑ Less than 12 years of schooling

❑ High school

❑ Certificate studies

❑ Bachelor's degree

❑       Master's degree

❑       doctorate

**🖍 Weight:** ________________

**🖍 Height:** ________________

**🖍 BMI**: ________________

**Recalling the medical treatment you received in the past:**

**🖍** I felt that the approach of the medical staff is **less respectful** because of my excess weight

1 – Never, 2 – Rarely, 3 – Sometimes, 4 – In most cases, 5 – All the time

**🖍** I felt that my overweight is **causing the medical staff discomfort**

1 – Never, 2 – Rarely, 3 – Sometimes, 4 – In most cases, 5 – All the time

**🖍** I felt that because of my excess weight I receive **less optimal treatment** from the medical staff

1 – Never, 2 – Rarely, 3 – Sometimes, 4 – In most cases, 5 – All the time

**🖍**During medical appointments I **have experienced insulting, insensitive and judgmental** approach related to my overweight

1 – Never, 2 – Rarely, 3 – Sometimes, 4 – In most cases, 5 – All the time

**🖍** If you have experienced insulting or disrespectful approach from the medical staff due to being overweight, we would appreciate it if you could mention **the profession of the staff member**:

- Family doctor

- Gynecologist

- Doctor specializing in orthopedics

- Anesthesiologist

- A nurse

- Medical secretary

- Imaging technician

- Psychologist

- Social worker

- Other

**🖍** If you have experienced insulting or disrespectful approach from the medical staff due to being overweight, we would appreciate if you could give a **prominent example/s** from your past: ________________

**🖍** During medical appointments it happened that I was told by the medical staff **to lose weight without me wanting to talk about** this at all:

1 – Never, 2 – Rarely, 3 – Sometimes, 4 – In most cases, 5 – All the time

**🖍** During medical appointments it happened that I was told by the medical staff **to lose weight** **without them knowing my medical background**

1 – Never, 2 – Rarely, 3 – Sometimes, 4 – In most cases, 5 – All the time

**🖍** During medical appointments it happened that I was told by the medical staff **to lose weight when the reason for my appointment was unrelated** to my weight:

1 – Never, 2 – Rarely, 3 – Sometimes, 4 – In most cases, 5 – All the time

**🖍** If relevant, we would appreciate if you could give **an example** of a situation where you have been told by the medical staff to lose weight when the reason for the appointment was unrelated to your weight: __________

**🖍** During medical appointments it happened that I was told by the medical staff to lose weight **without giving effective and practical tools on** how to do it

1 – Never, 2 – Rarely, 3 – Sometimes, 4 – In most cases, 5 – All the time

**🖍** It happened **that the office equipment** was not suitable for overweight people (chairs without handles, a wide sphygmomanometer, etc.).

1 – Never, 2 – Rarely, 3 – Sometimes, 4 – In most cases, 5 – All the time

If relevant, we would appreciate if you could give an example of a situation in which **the office equipment**was not adapted for overweight people: ______________________

**🖍** Have you ever **felt frustrated** due to the medical staff's approach to overweight people?

1 – Never, 2 – Rarely, 3 – Sometimes, 4 – In most cases, 5 – All the time

**🖍** Have you ever **avoided a needed appointment** with a doctor due to fear of disrespectful treatment because of excess weight?

1 – Never, 2 – Rarely, 3 – Sometimes, 4 – In most cases, 5 – All the time

**Please mark the degree of your agreement with the following statements:**

**🖍** I agree that overweight is **a risk factor for various** **medical problems**

1 – Not at all, 2 – To a small extent, 3 – To a moderate extent, 4 – To a large extent, 5 - Absolutely

**🖍** **I would like to lose weight**

1 – Not at all, 2 – To a small extent, 3 – To a moderate extent, 4 – To a large extent, 5 - Absolutely

**🖍** I believe that **the medical staff should refer** to the issue of overweight of their patients

1 – Not at all, 2 – To a small extent, 3 – To a moderate extent, 4 – To a large extent, 5 - Absolutely

**🖍** I would prefer that the medical staff **would not raise the issue** of my weight:

1 – Not at all, 2 – To a small extent, 3 – To a moderate extent, 4 – To a large extent, 5 - Absolutely

**🖍** I would prefer that the medical staff **would raise the issue** of my weight **only in case it is associated with the medical problem** I came to treat:

1 – Not at all, 2 – To a small extent, 3 – To a moderate extent, 4 – To a large extent, 5 - Absolutely

**🖍** I would prefer that the medical staff **would ask me in advance** whether I agree to talk about the issue of my overweight

1 – Not at all, 2 – To a small extent, 3 – To a moderate extent, 4 – To a large extent, 5 - Absolutely

**🖍** I feel that the medical staff **does not understand the difficulty**in losing weight

1 – Not at all, 2 – To a small extent, 3 – To a moderate extent, 4 – To a large extent, 5 - Absolutely

**🖍** I would prefer that the medical staff w**ould offer me effective and practical tools** to lose weight

1 – Not at all, 2 – To a small extent, 3 – To a moderate extent, 4 – To a large extent, 5 - Absolutely

**🖍**What would you like to **say to the medical staff**regarding their approach to people with overweight?

____________________________________________________________________________________________________________________________________________________________________________________________________

**🖍**In your opinion, **what can be done to improve** the medical staff's attitude towards overweight patients?

**🖍 Additional comments** (if any):_______________________________________

____________________________________________________________________________________________________________________________________________________________________________________________________
