# Supplementary material for: Experiences of weight stigmatization in the Israeli healthcare system among overweight and obese individuals
Source: Isr J Health Policy Res. 2022 Jan 31;11:5. doi: 10.1186/s13584-022-00518-9 (PMC8802507; doi:10.1186/s13584-022-00518-9)
Supplement: Supplementary file 2 — Additional file 2. Univariate analysis of the correlation of weight categories to adverse feelings and experiences. [file 13584_2022_518_MOESM2_ESM.docx]

Supplementary Table 2. Univariate analysis of the correlation of weight categories to adverse feelings and experiences.

|  | **Overweight**  **n=267** | **Class I obesity**  **n=495** | | **Class II obesity**  **n=479** | | **Class III obesity**  **n=456** | |
| --- | --- | --- | --- | --- | --- | --- | --- |
|  | **% (ref)** | **%** | RR (95%CI) | **%** | RR (95%CI) | **%** | RR (95%CI) |
| Disrespectful treatment due to overweight | **43.2** | **55.0** | 1.2 (1.1-1.3) | **59.8** | 1.3 (1.1-1.4) | **71.6** | 1.6 (1.4-1.8) |
| A feeling of discomfort of the personnel due to overweight | **23.0** | **36.2** | 1.2 (1.1-1.4) | **45.1** | 1.4 (1.3-1.5) | **62.6** | 1.8 (1.6-2.0) |
| Having received less optimal treatment | **32.7** | **43.9** | 1.2 (1.1-1.3) | **49.9** | 1.3 (1.2-1.4) | **61.6** | 1.5 (1.4-1.7) |
| Experience of insulting, insensitive and judgmental approach | **41.7** | **54.4** | 1.2 (1.1-1.3) | **62.2** | 1.4 (1.2-1.5) | **67.0** | 1.5 (1.3-1.7) |
| Office equipment not suitable for overweight people | **12.1** | **20.6** | 1.2 (1.1-1.3) | **28.0** | 1.3 (1.2-1.5) | **49.1** | 1.7 (1.5-1.9) |
| Avoidance of appointment due to fear of disrespectful treatment | **26.0** | **33.4** | 1.1 (1.01-1.3) | **43.6** | 1.3 (1.2-1.4) | **53.2** | 1.5 (1.3-1.7) |

BMI categories: overweight (25-29.9 kg/m^2^), class I obesity (30-34.9 kg/m^2^), class II obesity (35-39.9 kg/m^2^), and class III obesity (BMI of 40 kg/m^2^ or greater)
